# P-918. Antibiotic Stewardship Initiative for Adults with Uncomplicated Community Acquired Pneumonia

**DOI:** 10.1093/ofid/ofaf695.1124

**Published:** 2026-01-11

**Authors:** Shuchi Amin, Andrea Call, Jill Bellows, Christopher Noel, Joseph Miles, Ivayla I Geneva

**Affiliations:** SUNY Upstate Medical University, Syracuse, NY; Crouse Hospital, Syracuse, New York; Crouse Hospital, Syracuse, New York; Crouse Hospital, Syracuse, New York; Crouse Hospital, Syracuse, New York; Crouse Hospital, Syracuse, New York

## Abstract

**Background:**

Community acquired pneumonia (CAP) is a leading cause of morbidity and mortality in the US with an annual incidence of 248 per 100,000 adults and 6% mortality when hospitalized. The Infectious Diseases Society of America’s clinical guidelines recommend a minimum of 5 days antibiotic treatment for uncomplicated CAP showing clinical improvement. Antibiotic overuse and the associated development of antibiotic resistance is concerning and a promising target for antibiotics stewardship is the management of uncomplicated CAP with shorter antibiotic courses.

**Figure 1.**
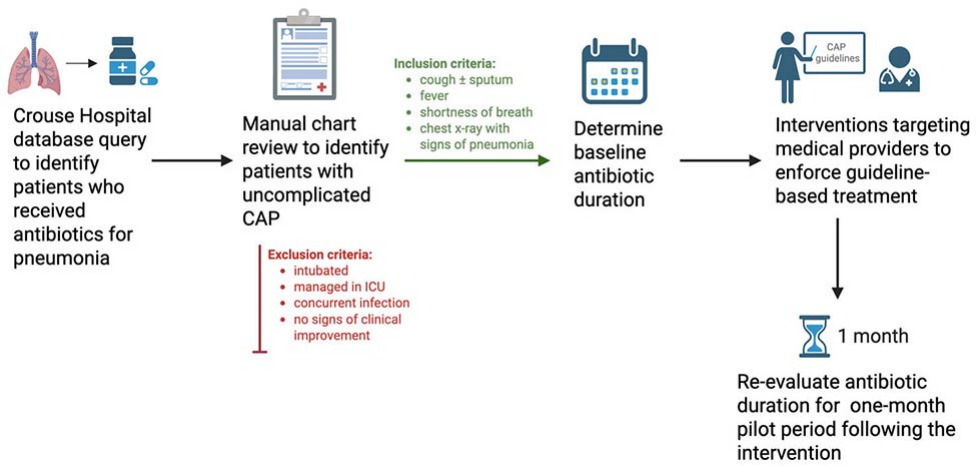
Methods for identification of patients with community acquired pneumonia, determination of antibiotic course length, implementation of provider-targeted interventions to enforce guideline-driven antibiotic duration, and subsequent re-evaluation of antibiotic course length following a one-month pilot period.

**Figure 2.**
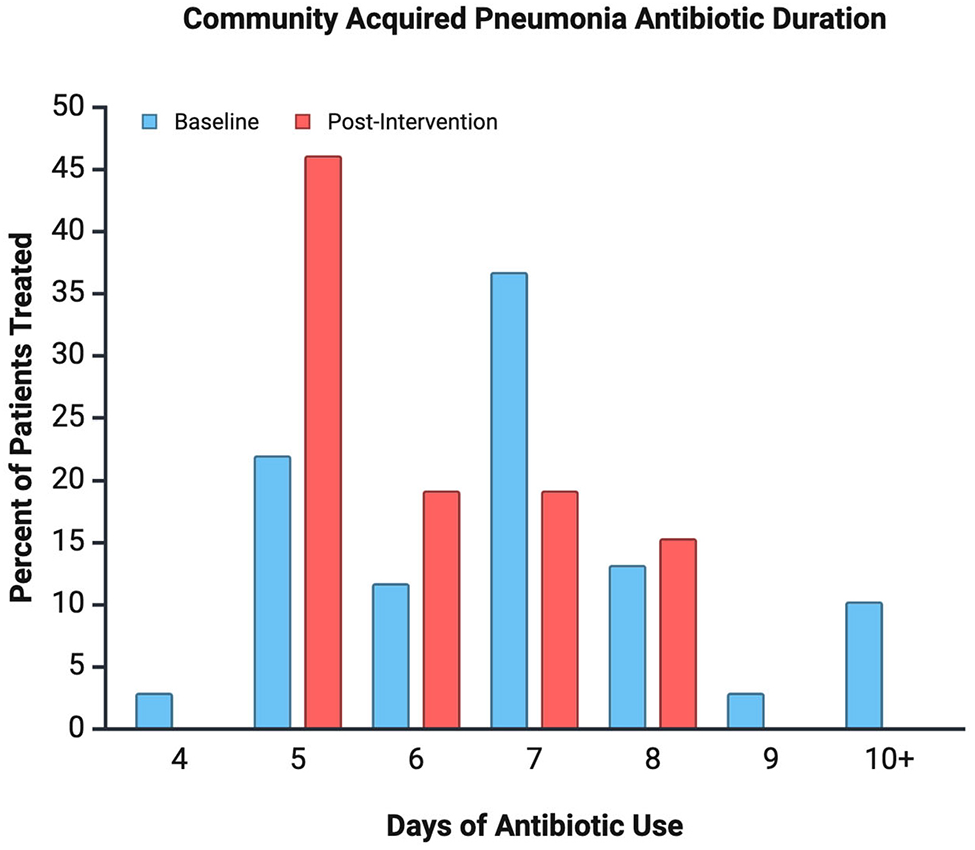
Antibiotic duration for patients admitted to a tertiary hospital (Crouse Hospital, Syracuse, NY) for community acquired pneumonia (CAP). Data comparison is shown between a one-month baseline antibiotic duration in December 2024 (blue) and a one-month post-intervention period in March 2025 (red). Interventions included provider-targeted measures of disseminating inclusion and exclusion criteria for CAP as well as contacting prescribers on day 3 of treatment to encourage guideline-recommended 5 day courses for patients with clinical improvement. Baseline antibiotic duration was 6.97 ± 1.89 days and post-intervention antibiotic duration was 6.04 ± 1.15 days, showing 47.2% better adherence to IDSA guidelines.

**Methods:**

This is the pilot portion of a quality improvement study of patients admitted to a tertiary medical center (Crouse Hospital in Syracuse, NY) from December 2024 to April 2025. We performed a database query to identify patients who received antibiotics for pneumonia and, using manual chart review, identified those who met the following criteria for uncomplicated CAP: cough ± sputum, fever, shortness of breath, and chest x-ray showing consolidation, ground-glass opacities or infiltrates. We excluded patients who were intubated, in the ICU, had additional infections, or lacked signs of clinical improvement. For patients meeting our criteria, we noted the baseline duration of antibiotic courses. We then implemented provider-targeted interventions to support guideline-based treatment. This included disseminating our inclusion and exclusion criteria and contacting prescribers on day 3 of treatment to encourage 5-day courses. We evaluated antibiotic duration for the one-month pilot period following the intervention with a primary objective of decreasing the overuse of antibiotics by 25%.

**Results:**

From the 203 patients treated with antibiotics for pneumonia before the educational intervention, 68 met our inclusion and exclusion criteria. The baseline antibiotics duration was 6.97 ± 1.89 days (standard deviation). During the month following the intervention, there were 66 patients treated for pneumonia and 26 met our criteria, yielding an average antibiotic duration of 6.04 ± 1.15 days.

**Conclusion:**

Prior to intervention, antibiotics courses were higher than guidelines’ recommendations of 5 days. The pilot-based data shows a decrease in antibiotic overuse by 47.2% with better adherence to the IDSA guidelines.

**Disclosures:**

All Authors: No reported disclosures

